# The *PNPLA3* rs738409 148M/M Genotype Is a Risk Factor for Liver Cancer in Alcoholic Cirrhosis but Shows No or Weak Association in Hepatitis C Cirrhosis

**DOI:** 10.1371/journal.pone.0027087

**Published:** 2011-11-07

**Authors:** Hans Dieter Nischalke, Cordula Berger, Carolin Luda, Thomas Berg, Tobias Müller, Frank Grünhage, Frank Lammert, Martin Coenen, Benjamin Krämer, Christian Körner, Natascha Vidovic, Johannes Oldenburg, Jacob Nattermann, Tilman Sauerbruch, Ulrich Spengler

**Affiliations:** 1 Department of Internal Medicine I, University of Bonn, Bonn, Germany; 2 Department of Gastroenterology, University Hospital Leipzig, Leipzig, Germany; 3 Medical Clinic for Hepatology and Gastroenterology, Medical University Charité Campus, Virchow–Klinikum Berlin, Berlin, Germany; 4 Department of Internal Medicine II, Saarland University Hospital, Homburg, Germany; 5 Institute for Experimental Hematology and Transfusion Medicine, University of Bonn, Bonn, Germany; Broad Institute of Massachusetts Institute of Technology and Harvard University, United States of America

## Abstract

**Background:**

An isoleucine>methionine mutation at position 148 in the PNPLA3 gene (p.I148M, rs738409) has recently been identified as a susceptibility factor for liver damage in steatohepatitis. Here, we studied whether the *PNPLA3* rs738409 polymorphism also affects predisposition to hepatocellular carcinoma (HCC).

**Methods:**

We compared distributions of *PNPLA3* genotypes in 80 and 81 Caucasian patients with alcoholic and hepatitis C virus (HCV)-associated HCC to 80 and 81 age- and sex-matched patients with alcohol-related and HCV-related cirrhosis without HCC, respectively. *PNPLA3* genotypes in 190 healthy individuals from the same population served as reference. Potential confounders obesity, diabetes, HCV genotype and HBV co-infection were controlled by univariate and multivariate logistic regression with forward variable selection.

**Results:**

*PNPLA3* genotypes were in Hardy-Weinberg equilibrium for all study groups. The frequency of the 148M allele was significantly (p<0.001) increased in alcoholic cirrhosis with (53.7%) and without HCC (36.2%) but was not different between healthy controls (22.9%) and patients with cirrhosis (25.3%; p = 0.545) and HCC (30.2%; p = 0.071) due to hepatitis C. HCC risk was highest in 148M/M homozygous patients with alcoholic liver disease (odds ratio (OR) 16.8 versus healthy controls; 95% confidence interval (CI) 6.68–42.43, p<0.001). Finally, multivariate regression confirmed 148M/M homozygosity (OR 2.8; 95%-CI: 1.24–6.42; p = 0.013) as HCC risk factor in alcoholic cirrhosis. In HCV-related cirrhosis only HCV genotype 1 was confirmed as a HCC risk factor (OR 4.2; 95%-CI: 1.50–11.52; p = 0.006).

**Conclusion:**

The *PNPLA3* 148M variant is a prominent risk factor for HCC in patients with alcoholic cirrhosis, while its effects are negligible in patients with cirrhosis due to HCV. This polymorphism provides an useful tool to identify individuals with particularly high HCC risk in patients with alcoholic liver disease that should be taken into account in future HCC prevention studies.

## Introduction

Hepatocellular carcinoma (HCC) is a leading cause of cancer-related death worldwide [1], [2]. HCC is mainly attributed to chronic viral hepatitis B and C in developing countries [3], whereas in Europe and North America approximately 45% of HCCs are caused by increased alcohol consumption [4].

Great interest has come from a genome-wide association study that identified a single-nucleotide polymorphism (rs738409C/G) in the *PNPLA3* gene on chromosome 22, encoding an isoleucine→methionine substitution (p.I148M) of patatin-like phospholipase A3, also termed adiponutrin as risk factor of steatohepatitis and liver cirrhosis in alcoholic and non-alcoholic fatty liver disease [5], [6]. Adiponutrin is a transmembrane protein expressed in human adipose tissue and liver. Although its precise in vivo function is still unknown, current data indicate a pivotal role in lipid homeostasis. Adiponutrin expression is down-regulated during fasting and is induced during high calorie intake because gene activity is up-regulated in response to glucose, insulin, and thyroid hormones. Thus, adiponutrin is an important regulator of hepatic lipid metabolism during nutritional excess [6], [7]. However, this genetic variant was not correlated to body mass index, visceral or subcutaneous fat content, insulin sensitivity or peripheral blood lipid levels [8], [9]. Furthermore, it correlated inversely with carotid artery intima thickness, suggesting that the I148M polymorphism selectively affects fat deposition in the liver but is not linked to a general metabolic disorder [6], [10], [11]. However, the 148M variant was correlated with raised serum alanine aminotransferase levels [12], [13], [14] elevated hepatic fat content and increased rates of fibrosis in alcoholic and non-alcoholic fatty liver disease [15], [16]. Here, we studied if the adiponutrin 148M allele had any effects on the risk of HCC development among cirrhotic patients with alcoholic liver disease as compared to chronic viral hepatitis C, an alternative strong risk factor for HCC.

## Materials and Methods

### Ethics Statement

The reported studies were approved by the Institutional Review Boards of the Bonn and Berlin University Ethics Committees. Written informed consent was obtained from the patients prior to sample collection. Samples were coded and data stored anonymously.

### Study groups

We recruited 161 patients with hepatocellular carcinoma (HCC) at the Bonn and Berlin University Departments of Gastroenterology between 2005 and 2009. In 80 patients HCC was related to alcoholic cirrhosis and in 81 patients to chronic hepatitis C. These HCC patients were compared to 80 and 81 sex and age (±3 years)-matched patients with alcoholic cirrhosis and HCV-associated cirrhosis, who did not have liver cancer. Cirrhotic patients without liver cancer had at least one year of follow-up to guard against the possibility of occult malignancy. 190 healthy volunteers from the same background population served as a reference. Patients were considered to have alcoholic cirrhosis if their history indicated average alcohol consumption to exceed 300 g ethanol per week. Patients with mixed HCV infection and increased alcohol consumption (>300 g/week) had been excluded from this study. The distribution of HCV genotypes was 90.0% genotype 1, 1.3% genotype 2, 7.5% genotype 3, 1.3% genotype 4 in patients with liver cancer and 61.7% genotype 1, 22.2% genotype 2, 8.6% genotype 3, 7.4% genotype 4 in HCV-infected cirrhotic patients without HCC. All subjects in this study were Caucasians. Further demographic and clinical characteristics are listed in table 1.

**Table 1:** Demographic and Clinical Data of the Study Groups.

	Alcoholic cirrhosis with HCC (n = 80)	Alcoholic cirrhosis without HCC (n = 80)	HCV cirrhosis with HCC (n = 81)	HCV cirrhosis without HCC (n = 81)	healthy controls (n = 190)
**Median age**, years (range)	**56 (39–80)**	**56 (39–81)**	**56 (38–82)**	**56 (37–82)**	**40.5 (20–86)**
**Gender** (% male/female)	**86/14**	**86/14**	**57/43**	**57/43**	**56/44**
**Alcohol abuse** (%)	**100**	**100**	**0**	**0**	**-**
**HCV** (%)	**0**	**0**	**100**	**100**	**-**
**HBV** (%)	**5.0**	**3.8**	**8.6**	**1.2**	**-**
**BMI>30**, [kg/m^2^] (%)	**20.0**	**30.0**	**29.6**	**27.2**	**-**
**Diabetes** (%)	**18.8**	**26.3**	**11.1**	**18.5**	**-**
**Child-Pugh class** A/B/C (%)	**47.5/30.0/22.5**	**3.8/47.5/48.8**	**55.6/30.8/13.6**	**56.8/28.4/14.8**	**-**
**Platelet count** [*10^3^/µl], (Mean ± SD)	**139±57**	**149±82**	**114±58**	**148±130**	**-**
**ALT** [IU/l], (Mean ± SD)	**49±39**	**59±241**	**121±231**	**97±76**	**-**
**AST** [IU/l], (Mean ± SD)	**93±114**	**129±430**	**112±123**	**84±59**	**-**
**GGT** [IU/l], (Mean ± SD)	**196±158**	**202±215**	**99±90**	**146±136**	**-**

All patients underwent careful clinical examination, standard laboratory tests and abdominal ultrasound. Chronic viral hepatitis was diagnosed by routine testing for hepatitis B surface antigen, HBV-DNA, HCV-RNA and HCV antibodies, respectively. Serum levels of ferritin, transferrin saturation, if necessary HFE genetic testing as well as quantitative immunoglobulins and autoantibodies were determined to exclude other etiologies of liver disease. Cirrhosis was diagnosed either by liver biopsy, transient elastography (stiffness>15 kPa), and signs of portal hypertension (splenomegaly, oesophageal varices, ascites). The diagnosis of HCC was made by contrast enhanced magnetic resonance imaging and computed tomography according to recently established diagnostic criteria [EASL 2009 und AASLD 2010 guidelines].

### Determination of rs738409 p.I148M alleles

Genomic DNA was extracted from 200 µL EDTA-blood using the QIAamp Blood Mini Kit (Qiagen, Hilden, Germany) according to the manufacturer's protocol. Determination of the *PNPLA3* rs738409 polymorphism was performed by LightCycler real time PCR (Roche, Mannheim, Germany) using a commercial LightSNiP (SimpleProbe) assay from TIB-MolBiol (Berlin, Germany) according to the manufacturer's recommendations.

### Statistical analysis

Genotype frequencies were determined and tested for consistency with the Hardy-Weinberg equilibrium using an exact test. Allele and genotype frequencies were compared between cases and controls by Pearson's goodness-of-fit chi^2^ test and Armitage's trend test, respectively (http://ihg2.helmholtz-muenchen.de/cgi-bin/hw/hwa1.pl). Differences between groups were analyzed by t-test and Mann-Whitney-U test as appropriate.

Power calculations were done using Lenth, R. V. (2006–9) Java Applets for Power and Sample Size [Computer software], retrieved Aug 25th, 2011 from http://www.stat.uiowa.edu/~rlenth/Power. Power calculation was targeted to ensure 80% statistical power at 5% alpha error.

To take into account further potential confounding risk factors of liver cancer the effects of HCV-genotype, HBV infection, diabetes and obesity were assessed by univariate comparisons (ANOVA and chi^2^-statistics) followed by multivariate logistic regression with forward variable selection. This analysis was performed separately in patients with alcohol- and HCV-related liver disease. Parameters with effects at p<0.1 were entered into the multivariate analysis with P<0.05 for inclusion and p>0.1 for exclusion as selection criterion for parameters in the final statistical models.

Statistical analysis was performed with SPSS 18.0 (SPSS, Munich, Germany). Data are reported as mean ± standard deviation, unless stated otherwise.

## Results

### Study population

Obesity and diabetes occurred more frequently in alcoholic cirrhosis without HCC than in alcoholic patients with HCC (table 1; p<0.05). Significantly higher GGT values where observed in the two groups with alcoholic liver disease than in patients with HCV infection (p<0.05), while ALT levels were significantly higher in the patients with hepatitis C (p<0.01 each). In patients with HCV-associated liver cirrhosis distribution of Child-Pugh classes was equivalent between patients with and without HCC. In contrast, Child Pugh classes B and C prevailed in the control group with alcohol-induced cirrhosis without HCC (p<0.001).

### *PNPLA3 (r*s738409) gene polymorphism and liver cancer

**Figure 1:**
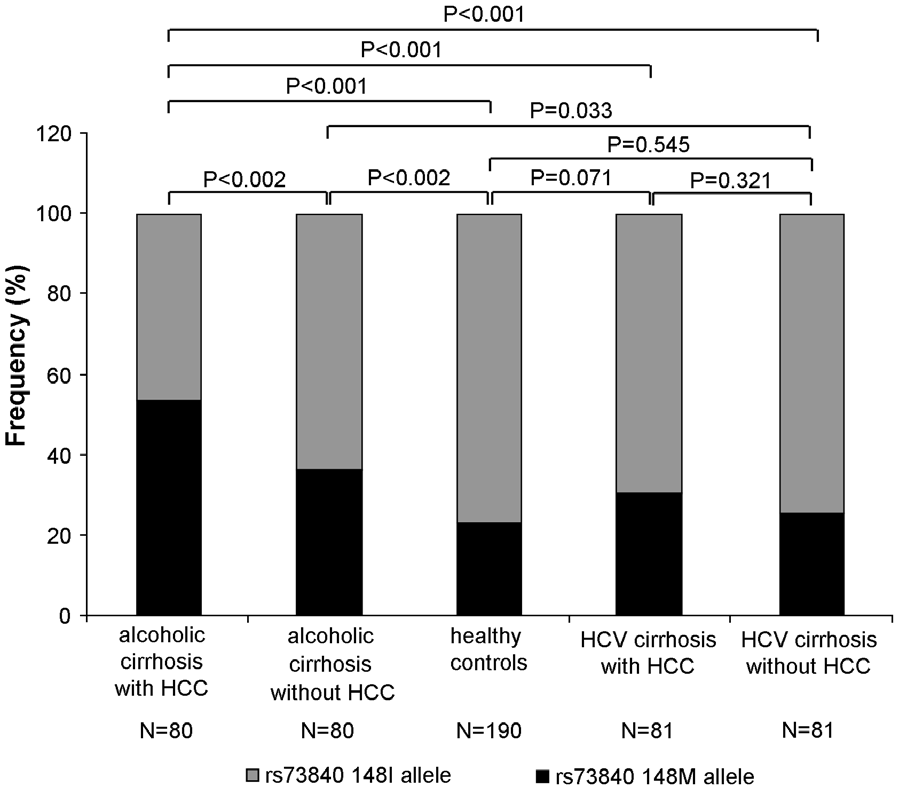
Frequencies of the *PNPLA3* rs738409 alleles in the study groups. This figure illustrates the frequencies of the 148I (grey part of columns) and 148M (dark part of columns) alleles of the *PNPLA3* (rs738409) polymorphism in patients with HCV-associated and alcohol-related HCC, patients with alcoholic and HCV-related cirrhosis, who do not have liver cancer, and healthy controls, respectively. Differences between the groups were compared by chi^2^-statistics.

The distributions of *PNPLA3* p.I148M alleles were consistent with the Hardy Weinberg equilibrium in all study groups (table 2). Of note, the distribution of *PNPLA3* I148M alleles was similar in healthy controls and patients with HCV-associated cirrhosis and HCV-related HCC giving rise to 22.9%, 25.3% (p = 0.545) and 30.2% (p = 0.071) frequencies of the 148M allele, respectively. In contrast, the prevalence of patients carrying a *PNPLA3* 148M variant was significantly increased in the two groups with alcoholic liver disease and reached allele frequencies of 53.7% (p<0.001 versus healthy controls) and 36.2% (p = 0.033 versus healthy controls) in patients with and without hepatocellular carcinoma, respectively (figure 1).

**Table 2:** Distribution of *PNPLA3* rs738409 (I148M) variants.

*PNPLA3* (I148M) Variants	alcoholic cirrhosis with HCC (n = 80)	alcoholic cirrhosis without HCC (n = 80)	healthy controls (n = 190)	HCV cirrhosis with HCC (n = 81)	HCV cirrhosis without HCC (n = 81)
**Genotype 148 I/I**	17 (21.2%)	32 (40.0%)	112 (58.9%)	40 (49.4%)	45 (55.6%)
**Genotype 148 I/M**	40 (50.0%)	38 (47.5%)	69 (36.3%)	33 (40.7%)	31 (38.3%)
**Genotype 148 M/M**	23 (28.8%)	10 (12.5%)	9 (4.7%)	8 (9.9%)	5 (6.2%)
**148M allele frequency**	56.5%	36.2%	22.9%	30.3%	25.3%
**Deviation from Hardy-Weinberg equilibrium (Exact test)**	p = 0.67	p = 1.0	p = 0.84	p = 0.79	p = 1.0

**Significance and odds ratios for the heterozygous genotype (I/M)**.

Alcoholic cirrhosis with HCC versus HCV/HCC:   2.852; 95%-CI (1.373–5.925); p<0.005.

Alcoholic cirrhosis with HCC versus HCV cirrhosis without HCC: 3.416; 95%-CI (1.648–7.080); p<0.001.

Alcoholic cirrhosis with HCC versus healthy controls:  3.819; 95%-CI (2.010–7.257); p<0.001.

Alcoholic cirrhosis without HCC versus healthy controls:  1.928; 95%-CI (1.103–3.367); p = 0.020.

**Significance and odds ratios for the homozygous genotype (M/M)**.

Alcoholic cirrhosis with HCC versus alcoholic cirrhosis without HCC: 4.329; 95%-CI (1.679–11.16); p<0.002.

Alcoholic cirrhosis with HCC versus HCV/HCC:   6.765; 95%-CI (2.528–18.11); p<0.001.

Alcoholic cirrhosis with HCC versus HCV cirrhosis without HCC: 12.18; 95%-CI (3.987–37.19); p<0.001.

Alcoholic cirrhosis with HCC versus healthy controls:  16.84; 95%-CI (6.682–42.43); p<0.001.

Alcoholic cirrhosis without HCC versus healthy controls:  3.889; 95%-CI (1.456–10.39); p<0.005.

**Significance and odds ratios for the minor allele frequencies**.

Alcoholic cirrhosis with HCC versus alcoholic cirrhosis without HCC: 2.281; 95%-CI (1.466–3.551); p<0.001.

Alcoholic cirrhosis with HCC versus HCV/HCC:   2.680; 95%-CI (1.697–4.233); p<0.001.

Alcoholic cirrhosis with HCC versus HCV cirrhosis without HCC: 3.430; 95%-CI (2.141–5.494); p<0.001.

Alcoholic cirrhosis with HCC versus healthy controls:  4.369; 95%-CI (2.969–6.429); p<0.001.

Alcoholic cirrhosis without HCC versus HCV cirrhosis without HCC: 1.678; 95%-CI (1.040–2.709); p = 0.033.

Alcoholic cirrhosis without HCC versus healthy controls:  1.915; 95%-CI (1.282–2.861); p<0.002.

The distributions of *PNPLA3* p.I148M genotypes are summarized in table 2: Patients with alcohol-related HCC comprised significantly more 148I/M heterozygous and 148M/M homozygous individuals than the healthy controls and the two groups of patients with alcoholic cirrhosis without liver cancer. The frequency of the *PNPLA3* 148M allele was increased both in alcohol-induced cirrhosis (OR = 1.92; 95%-CI: 1.28–2.86; p<0.002) and alcohol-associated liver cancer (OR = 4.37; 95%-CI: 2.97–6.43; p<0.001) as compared to healthy controls. In patients with alcoholic cirrhosis the risk of HCC was particularly linked to the homozygous 148M/M genotype (OR = 16.84; 95%-CI: 6.68–42.43; p<0.001). When we compared the risk of the PNPLA3 I148M polymorphism between alcoholic patients with and without HCC, differences in the allele frequency (OR = 2.28; 95%-CI: 1.47–3.55; p<0.001) and the frequency of the homozygous 148M/M genotype (OR = 4.33; 95%-CI: 1.68–11.16; p<0.002) still indicated a significant effect of the 148M allele on progression of alcoholic liver cirrhosis towards liver cancer. We could not find any significant associations between the PNPLA3 I148M polymorphism and serum levels of liver enzymes or markers of the metabolic syndrome (obesity, diabetes mellitus or serum lipids) (data not shown).

To exclude the possibility that the observed differences in the distribution of Child-Pugh classes might have affected our analysis, we stratified patients into the two classes A/B versus C. Then, we matched our alcoholic patients for sex, age and the dichotomized Child – Pugh class. After matching 42 pairs remained available for a supplementary analysis: In these matched pairs of alcoholic patients with and without hepatocellular carcinoma the PNPLA3 148M allele frequencies were 53.6% vs. 31.0% (OR = 2.57; 95%-CI: 1.37–4.84; p = 0.003), respectively. Likewise, frequencies of the homozygous 148M/M genotype were 31.0% vs 9.5% in matched patients with and without HCC (OR = 6.50; 95%-CI: 1.68–25.16; p = 0.004).

Finally we checked if the *PNPLA3* p.I148M polymorphism remained an independent HCC risk factor when other known risk factors of HCC such as obesity, diabetes, and HBV infection were also taken into account. Including all patients with alcohol- and HCV-induced liver disease, respectively, we calculated separate Cox regression models to identify HCC risk factors in patients with alcoholic cirrhosis (table 3) and HCV-associated cirrhosis (table 4). Homozygous *PNPLA3* 148M/M genotype (OR 2.83; 95%-CI: 1.24–6.42; p = 0.013) was confirmed as a risk factor of HCC in alcoholic cirrhosis. Further, HCV genotype 1 (OR 4.16; 95%-CI: 1.50–11.52; p = 0.006) was confirmed as a risk factor of HCC in HCV-associated cirrhosis.

**Table 3:** Regression analysis of putative HCC risk factors in alcoholic cirrhosis.

Initial Covariates (univariate comparisons)				
Parameter	P	OR	Lower	Upper
Diabetes mellitus	0.274	0.658	0.311	1.396
HBV infection	0.699	1.351	0.292	6.239
Obesity (BMI>30)	0.279	0.559	0.193	1.616
*PNPLA3* 148MM genotype	0.011	2.825	1.243	6.417

Parameters that remained in the final model (multivariate analysis*)				
Parameter	P	OR	Lower	Upper
*PNPLA3* 148MM genotype	0.013	2.825	1.243	6.417
Constant	0.250	0.814	-	-

*including all significant parameters from the univariate analysis (*PNPLA3* 148MM genotype).

CI, confidence interval; OR, odds ratio.

**Table 4:** Regression analysis of putative HCC risk factors in HCV-related cirrhosis.

Initial Covariates (univariate comparisons)				
Parameter	P	OR	Lower	Upper
Diabetes mellitus	0.174	0.542	0.222	1.321
HBV infection	0.031	7.473	0.898	62.21
HCV genotype 1	0.005	4.046	1.465	11.18
Obesity (BMI>30)	0.817	1.104	0.478	2.545
*PNPLA3* 148MM genotype	0.386	1.666	0.521	5.327

Parameters that remained in the final model (multivariate analysis*)				
Parameter	P	OR	Lower	Upper
HCV genotype 1	0.006	4.162	1.504	11.52
Constant	0.014	0.316	-	-

*including all significant parameters from the univariate analysis (HBV infection, HCV genotype).

CI, confidence interval; OR, odds rati.

## Discussion

Chronic hepatitis C and alcohol consumption are the leading causes for HCC in European populations. Here, we performed a cross-sectional analysis to study the potential role of the *PNPLA3* p.I148M variant in liver cancer associated with alcoholic liver disease as compared to patients with HCV-induced HCC. We found a steadily increasing prevalence of the 148M allele in patients with alcoholic cirrhosis (36.2%) and alcohol-associated HCC (53.7%) as compared to healthy controls (22.9%), whereas differences in the prevalence of this genetic variant between healthy controls and HCV-associated cirrhosis (25.3%) and HCV-related HCC (30.2%) were minor and not statistically significant. These findings are in line with previous observations in alcoholic liver disease and strengthens the concept that the PNPLA3 148M variant is associated with more severe liver damage and cirrhosis in alcoholic liver disease [17], [18]. Here, we add the novel observation that this genetic variant is also a strong genetic risk factor for alcohol-associated HCC, which contributes to an approximately 4-fold increased risk in homozygous carriers of the 148M allele even in the presence of established cirrhosis. This idea is further emphasized by our multivariate regression model, which confirmed *PNPLA3* 148M homozygosity as independent risk factors for HCC among our patients with alcoholic liver disease. On the other hand, the *PNPLA3* 148M variant had only minor effects on the HCC risk in cirrhosis associated with chronic hepatitis C suggesting that this genetic variant is not a tumour gene per se but only acts in combination with substantial alcohol exposure and hepatic lipid accumulation.

Two recent studies from Italy reported strong effects of the *PNPLA3* 148M variant also on the risk of HCC in chronic hepatitis C [19], [20], while a third Italian study suggested that this allele was predominantly associated with metabolic cirrhosis [21]. In line with this latter report, we found only little effect of the *PNPLA3* 148M variant on the risk of HCC in our patients with HCV infection, in whom bias from concomitant alcohol consumption had been excluded with great care. In our HCV-infected patients *PNPLA3* 148M/M homozygosity revealed an odds ratio of 1.666 for the risk of liver cancer (table 4), which failed to reach statistical significance with our sample size. Power calculation indicated the need of >670 patients per study group in order to detect statistical significance (p<0.05) for this putative association. On the other hand the observed discrepancies between the various studies may reflect inadvertent dietary differences between the study populations or probably the fact, that patients with combined HCV infection and alcohol intake (>300 g/week) had not sufficiently been taken into account. This explanation would be in line with the concept that hepatic lipid accumulation in chronic hepatitis C genotype 1 is linked to the presence of other additional metabolic factors such as diabetes and obesity [22]. Importantly, the effect attributable to the *PNPLA3* p.I138M polymorphism in nutritional cirrhosis was not correlated to age, gender, body mass index or diabetes in line with previous reports [8].

Finally, there was a marked imbalance in liver disease severity between patients with and without HCC among alcohol-induced cirrhosis. This imbalance possibly reflects the fact that patients with early alcohol-induced cirrhosis (Child Pugh class A) only infrequently present at hospital services, if HCC is absent. Thus, patients in the alcohol-related control group without HCC had significantly more advanced cirrhosis than patients with alcohol-induced cirrhosis and HCC. To check if this finding had biased our analysis, we also analyzed the subgroup of patients, who could be matched for Child-Pugh classes in addition to sex and age. In line with our hypothesis this supplementary analysis confirmed a significant association between the *PNPLA3* 148M allele - and in particular the *PNPLA3* 148M/M genotype - and HCC (OR = 6.50; 95%-CI: 1.68–25.16; p = 0.004).

The physiological role of the PNPLA3 protein is still unclear. The *PNPLA3* gene codes for an enzyme that exhibits both triglyceride hydrolase and transacylation activity in vitro [23]. The methionine substitution at position 148 disrupts triglyceride hydrolase activity [24], but PNPLA3 ablation in two different mouse strains had not resulted in hepatic lipid accumulation and liver damage under a variety of different diets [25]. On the other hand over-expression of *PNPLA3* 148M in mice increased hepatic lipid content favouring a gain-of-function mutation [24]. In addition, a variety of other intracellular interactions are also discussed as potential alternative pathogenic mechanisms [26]. In the context of non-alcoholic steatohepatitis the *PNPLA3* risk allele promotes hepatic lipid accumulation and severity of fibrosis [6], [8], [10], [27], [28]. Likewise, regular alcohol consumption causes liver damage via steatohepatitis. There is also evidence that liver biopsies from carriers of a *PNPLA3* 148M allele, in particular 148M homozygous subjects, exhibit greater hepatic inflammation and liver damage than patients with the wild type gene at the same extent of lipid deposition [27]. Thus, it may be increased hepatic inflammatory activity and fibrosis in response to lipid accumulation, which is associated with the *PNPLA3* 148M variant that enhances the risk for HCC in alcoholic cirrhosis.

Whatever the underlying mechanisms, the link between the PNPLA3 polymorphism and HCC in alcoholic liver disease appears strong enough to bear practical consequences: the presence of a *PNPLA3* 148M allele, and especially *PNPLA3* 148M homozygosity, may provide an easy tool to identify individuals at particular risk for HCC at all stages of alcoholic liver damage. Thus, future HCC prevention and treatment strategies should also take into account the combined effect of the *PNPLA3* p.I148M polymorphism and alcohol in patients with chronic liver disease.
